# Successful adjuvant bi-weekly gemcitabine chemotherapy for pancreatic cancer without impairing patients’ quality of life

**DOI:** 10.1186/1477-7819-11-3

**Published:** 2013-01-09

**Authors:** Yoichi Toyama, Seiya Yoshida, Ryota Saito, Hiroaki Kitamura, Norimitsu Okui, Ryo Miyake, Ryusuke Ito, Kyonsu Son, Teruyuki Usuba, Takuya Nojiri, Katsuhiko Yanaga

**Affiliations:** 1 Department of Surgery, The Jikei University Kashiwa Hospital, 163-1, Kashiwashita, Kashiwa City, Chiba Prefecture, 277-8567, Japan; 2 Department of Surgery, The Jikei University School of Medicine, 3-25-8, Nishishinbashi, Minato Ward, Tokyo, 105-8461, Japan

**Keywords:** Pancreatic cancer, Adjuvant chemotherapy, Bi-weekly gemcitabine, Mild dose-intensity, Survival prolongation, Patient’s quality of life, QLQ-C30 and QLQ-PAN26

## Abstract

**Background:**

Although adjuvant gemcitabine (GEM) chemotherapy for pancreatic cancer is standard, the quality of life (QOL) in those patients is still impaired by the standard regimen of GEM. Therefore, we studied whether mild dose-intensity adjuvant chemotherapy with bi-weekly GEM administration could provide a survival benefit with acceptable QOL to the patients with pancreatic cancer.

**Methods:**

After a phase I trial, an adjuvant bi-weekly 1,000 mg/m^2^ of GEM chemotherapy was performed in 58 patients with pancreatic cancer for at least 12 courses (Group A). In contrast, 36 patients who declined the adjuvant bi-weekly GEM chemotherapy underwent traditional adjuvant 5FU-based chemotherapy (Group B). Careful periodical follow-ups for side effects of GEM and disease recurrence, and assessment of patients’ QOL using the EORTC QOL questionnaire (QLQ-C30) and pancreatic cancer-specific supplemental module (QLQ-PAN26) were performed. Retrospectively, the degree of side effects, patients’ QOL, compliance rate, disease-free survival (DFS), and overall survival (OS) in Group A were compared with those in Group B.

**Results:**

No severe side effects (higher than Grade 2 according to the common toxicity criteria of ECOG) were observed, except for patients in Group B, who were switched to the standard GEM chemotherapy. Patients’ QOL was better in Group A than B (fatigue: 48.9 ± 32.1 versus 68.1 ± 36.3, nausea and vomiting: 26.8 ± 20.4 versus 53.7 ± 32.6, diarrhea: 21.0 ± 22.6 versus 53.9 ± 38.5, difficulty gaining weight: 49.5 ± 34.4 versus 67.7 ± 40.5, *P* < 0.05). Compliance rates in Groups A and B were 93% and 47%. There was a significant difference in the median DFS between both groups (Group A : B =12.5 : 6.6 months, *P* < 0.001). The median OS of Group A was prolonged markedly compared with Group B (20.2 versus 11.9 months, *P* < 0.005). For OS between both groups, univariate analysis revealed no statistical difference in 69-year-old or under females, and T1–2 factors, moreover, multivariate analysis indicated three factors, such as bi-weekly adjuvant GEM chemotherapy, T2 or less, and R0.

**Conclusions:**

Adjuvant chemotherapy with bi-weekly GEM offered not only the advantage of survival benefits but the excellent compliance with acceptable QOL for postoperative pancreatic cancer patients.

## Background

Pancreatic cancer is increasing rapidly worldwide and the prognosis is still quite poor even if the patient undergoes curative resection [1]. Compared to conventional 5-fluorouracil (5FU)-based chemotherapies [2], gemcitabine (GEM) showed improved prognosis of patients with non-resectable advanced pancreatic cancer in a randomized trial [3]. Subsequently, studies on adjuvant chemotherapy with 5FU and/or GEM for patients with resectable pancreatic cancer have been reported [4-9].

Thereafter, in our institution, adjuvant GEM chemotherapy with the standard regimen for patients with resectable pancreatic cancer had been used from 2001 to 2003. However, the compliance rate was very low, 38%, due to severe side effects, such as gastrointestinal discomforts and emaciation, even though the adjuvant chemotherapy was adjusted according to the reduction and extension criteria. The majority of pancreatic cancer patients, over 40 cases, failed to continue the adjuvant GEM chemotherapy with the standard regimen, and died without obtaining the beneficial effects of GEM. From the bitter experience of the low compliance rate in our institution, we hypothesized that the dose intensity of adjuvant GEM chemotherapy with the standard regimen was too heavy and harmful for people of Asian descent, and a milder dose-intensity of adjuvant GEM chemotherapy with bi-weekly administration might be more suitable and lead to prolongation of DFS and/or OS without impairing patients’ QOL.

Thereby, after a phase I study, we studied whether mild dose-intensity adjuvant chemotherapy with bi-weekly GEM administration could provide prolonged disease-free survival (DFS) and/or overall survival (OS) without impairing patients’ quality of life (QOL).

## Methods

After approval from the IRB (the ethics committee for biomedical research) in our university, a phase I study of adjuvant bi-weekly GEM chemotherapy was conducted in our institute using three different doses: a high dose (1,200 mg/m^2^), a medium dose (1,000 mg/m^2^), and a low dose (800 mg/m^2^). Each dose group consisted of at least six patients; the compliance rate in the high dose group was low, 47%, while the rates in the middle and low dose groups were 96% and 97%. Moreover, there was no significant difference in the side effects between the middle and low dose groups according to the common toxicity criteria of the ECOG. Consequently, the appropriate dose-intensity of adjuvant bi-weekly GEM chemotherapy was set at 1,000 mg/m^2^ in our institute. During a five-year period between 2004 and 2009, 128 patients with ductal pancreatic carcinomas were treated with surgery in our institution. A clinical study of mild dose-intensity, adjuvant bi-weekly GEM chemotherapy was performed with a total of 58 pancreatic cancer patients (Group A), who gave informed consent (IC). On the other hand, 36 patients (Group B), who did not give IC for adjuvant bi-weekly GEM chemotherapy, chose 5FU-based chemotherapy as the traditional adjuvant treatment. The remaining 34 patients were excluded from this study due to: macroscopic non-curative surgical treatment (22 cases), previous treatment of the current disease with more than one chemotherapeutic regimen and/or radiotherapy (3 cases), major complications after surgery such as aspiration pneumonia and/or leakage of pancreatojejunostomy (2 cases), hospital death after aggressive progression of the disease (1 case), not less than 85 years old (1 case), not providing IC for adjuvant chemotherapy (1 case), and physical conditions: active infection (1 case), interstitial pneumonia or pulmonary fibrosis (1 case), myocardial infarction within 3 months (1 case), and concomitant advanced cancer (1 case).

In Group A, the first administration of 1,000 mg/m^2^ of GEM was given during the third week after surgery, that is, 15–21 postoperative days (POD) if the patient’s condition was favorable. One cycle of this regimen was defined as twice bi-weekly administrations of GEM. After discharge from our hospital, administration of 1,000 mg/m^2^ of GEM was given consecutively to the outpatients at bi-weekly intervals, for at least six full cycles or until the patient’s condition was considered tolerable. If severe side effects occurred, the 1,000 mg/m^2^ dosage of GEM was reduced to 800 mg/m^2^. These severe side effects were: leukopenia <1,000 / mm^-3^, decreasing platelets <20,000 / mm^-3^, neutrophils <1,000 / mm^-3^ with fever (>38°C) or infection, and non-hematologic toxicity higher than Grade 3 according to the common toxicity criteria of the ECOG except for gastrointestinal toxicity, such as nausea, vomiting, diarrhea, and stomatitis. Additionally, when there were multiple side effects, the administration of GEM was extended until the patient’s recovery. If recovery needed more than two weeks, the study was discontinued. The multiple side effects were: leukopenia <2,000 / mm^-3^, decreasing platelets <70,000 / mm^-3^, and non-hematologic toxicity higher than Grade 2 according to the common toxicity criteria of the ECOG except for gastrointestinal toxicity, such as nausea, vomiting, diarrhea, and stomatitis.

In Group B, the postoperative patients were treated with adjuvant 5FU-based chemotherapy consisting of an intravenous 20 mg/m^2^ bolus of leucovorin followed by an intravenous 400 mg/m^2^ bolus of fluorouracil given on each 5 consecutive days every 28 days for 6 cycles. The adjuvant 5FU-based chemotherapy was also started during the third week after surgery, the same as Group A. When recurrence was identified in Group B, the adjuvant chemotherapy was converted from 5FU-based to standard GEM administration (1,000 mg/m^2^ on days 1, 8, and 15, repeated every four weeks for a total of at least six courses) as for the patients who had agreed with the therapy alteration.

The patients in both groups were followed up carefully, especially for side effects from the agents, disease recurrence, and the patients’ QOL, as measured according to the European Organisation for Research and Treatment of Cancer’s (EORTC) quality of life questionnaire (quality of life questionnaire – core 30 or QLQ-C30) and the pancreatic cancer-specific supplemental module (quality of life questionnaire – pancreatic cancer module 26 or QLQ-PAN26) [10-13]. The assessment of patients’ QOL in both groups was carried out at each outpatient appointment. Postoperative surveillance for the recurrence of pancreatic cancer was undertaken every three months.

Thus, the 58 cases in Group A were compared to the 36 cases in Group B retrospectively. Statistical analysis was performed using student’s t-test, the chi-square test, the Mann–Whitney test, the log-rank test, the Kaplan–Meier method, and the Cox hazard proportional model. Results were considered to be statistically significant when *P* < 0.05.

## Results

The patients’ ages in Group A ranged from 34 to 82 (median 68.0) years, the patients’ other characteristics such as sex, operative procedure, primary tumor size, nodal status, resection status, and the UICC’s TNM classification [14] are displayed in Table 1. Of the patients in Group A, 54 were able to receive every bi-weekly adjuvant chemotherapy treatment of 1,000 mg/m^2^ of GEM, although the other four patients needed an occasional extension of administration interval from bi- to tri-weekly, not because of hematologic toxicity but because of their suboptimal physical status.

**Table 1:** Characteristics of eligible patients

	**Group A (*n*= 58)**		**Group B (*n*= 36)**		***P*value**
	**Number**	**%**	**Number**	**%**	
Age (years)					
Median	68		69.5		0.07
Range	34–82		50–84		
Sex					
Male	36	62.1	20	55.6	0.32
Female	22	37.9	16	44.4	
Operative procedure					
PD^a^	45	77.6	26	72.2	0.36
DP^b^	13	22.4	10	27.8	
Primary tumor size					
T1	3	5.2	1	2.8	
T2	8	13.8	8	22.2	0.47
T3	36	62.1	21	58.3	
T4	11	19.0	6	16.7	
Nodal status					
N0	27	46.6	15	41.7	0.45
N1	31	53.4	21	58.3	
Resection status ^c^					
R0	38	65.5	22	61.1	0.49
R1	20	34.5	14	38.9	
UICC stage ^d^					
I	10	17.2	7	19.4	
II	27	46.6	15	41.7	0.38
III	7	12.1	4	11.1	
IV	14	24.1	10	27.8	
Histology					
Adenocarcinoma	54	93.1	33	91.7	0.69
Other	4	6.9	3	8.3	

^a^ Pancreatoduodenectomy, ^b^ distal pancreatectomy, ^c^ R0 or R1 means negative or positive margin microscopically, ^d^ sixth edition of International Union Against Cancer stages.

The patients’ age in Group B varied from 50 to 84 (median 69.5) years and the patients’ other characteristics are also indicated in Table 1. There were no statistical differences between both groups in the characteristics of the eligible patients. Except when during the implementation of the standard GEM administration as the second line of chemotherapy for the patients with recurrence disease in Group B, no severe side effects (higher than Grade 2 according to the common toxicity criteria of the ECOG) were observed. Ultimately, 30 cases of the 36 patients in Group B (83.3%) were converted from traditional 5FU-based chemotherapy to GEM administration with the standard regimen due to the recurrence of pancreatic cancer.

Only 11 patients in Group B were able to receive continuously the second line of standard GEM chemotherapy until they collapsed; accordingly, the compliance rate of the standard GEM chemotherapy in this study was low, 36.7% (11/30). Consequently, the total compliance rate in Group B was 47.2% (17/36). Compared with the low compliance rate in Group B, most of the patients in Group A continuously received the adjuvant bi-weekly GEM chemotherapy with a 93% (54/58) compliance rate until their acceptable physical status.

The QOL of patients in Group A was immensely better than that of patients in Group B, in which most of the patients converted to the standard GEM chemotherapy (*P* < 0.05), especially for fatigue, nausea and vomiting, diarrhea, and difficulty gaining weight in the symptom scales (Table 2).

**Table 2:** Assessment of patients’ quality of life

	**Group A (*n*= 58)**	**Group B (*n*= 36)**	***P*value**
Functional scales, mean ± SD			
Physical status	69.5 ± 31.3	67.8 ± 33.4	NS
Working ability	58.6 ± 40.1	55.4 ± 42.2	NS
Cognitive functioning	67.5 ± 34.8	66.1 ± 36.6	NS
Emotional functioning	62.3 ± 38.6	59.4 ± 28.5	NS
Social functioning	58.1 ± 32.5	53.3 ± 39.7	NS
Global quality of life	61.9 ± 33.3	51.7 ± 37.0	NS
Symptom scales, mean ± SD			
Fatigue	48.9 ± 32.1	68.1 ± 36.3	*P* < 0.05
Nausea and vomiting	26.8 ± 20.4	53.7 ± 32.6	*P* < 0.05
Pain	36.2 ± 28.5	34.2 ± 30.9	NS
Dyspnea	22.0 ± 27.9	32.2 ± 22.8	NS
Insomnia	55.7 ± 38.7	61.1 ± 41.7	NS
Appetite loss	33.3 ± 36.1	43.3 ± 39.1	NS
Constipation	23.8 ± 31.8	26.4 ± 30.4	NS
Diarrhea	21.0 ± 22.6	53.9 ± 38.5	*P* < 0.05
Financial difficulties	52.3 ± 37.3	58.8 ± 40.4	NS
Pancreas-specific pain	44.4 ± 29.2	49.5 ± 36.6	NS
Diet restriction	42.5 ± 32.0	54.1 ± 37.8	NS
Jaundice and pruritus	12.7 ± 20.1	18.8 ± 19.9	NS
Steatorrhea	36.1 ± 30.4	39.1 ± 34.7	NS
Poor body image	38.3 ± 34.2	59.8 ± 35.0	NS
Sexual dysfunction	66.8 ± 35.8	75.3 ± 41.1	NS
Dissatisfaction with care	61.2 ± 37.7	65.6 ± 39.1	NS
Bloating	41.7 ± 31.5	49.7 ± 37.4	NS
Bad-tasting food	29.4 ± 36.9	37.4 ± 38.2	NS
Indigestion	30.7 ± 30.1	39.5 ± 28.6	NS
Flatulence	35.7 ± 31.5	38.0 ± 32.3	NS
Difficulty gaining weight	49.5 ± 34.4	67.7 ± 40.5	*P* < 0.05
Weakness	45.9 ± 32.8	56.6 ± 42.7	NS
Dry mouth	40.5 ± 38.0	49.1 ± 36.9	NS
Treatment side-effects	39.1 ± 30.5	46.2 ± 33.8	NS
Worry about future	57.8 ± 35.8	66.3 ± 27.6	NS
Difficulty planning	34.6 ± 33.7	46.8 ± 45.2	NS

*SD* standard deviation; *NS* no significant difference.

**Figure 1:**
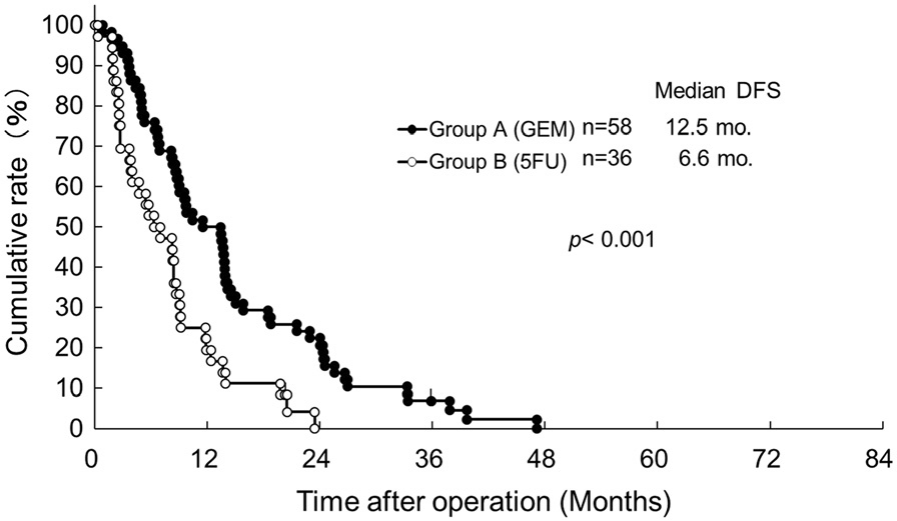
**Disease-free survival (DFS) of resected pancreatic cancer patients who received adjuvant chemotherapy with bi-weekly gemcitabine (GEM: Group A) versus 5FU-based (5FU: Group B).** mo., months (Log-rank test *P* < 0.001).

**Figure 2:**
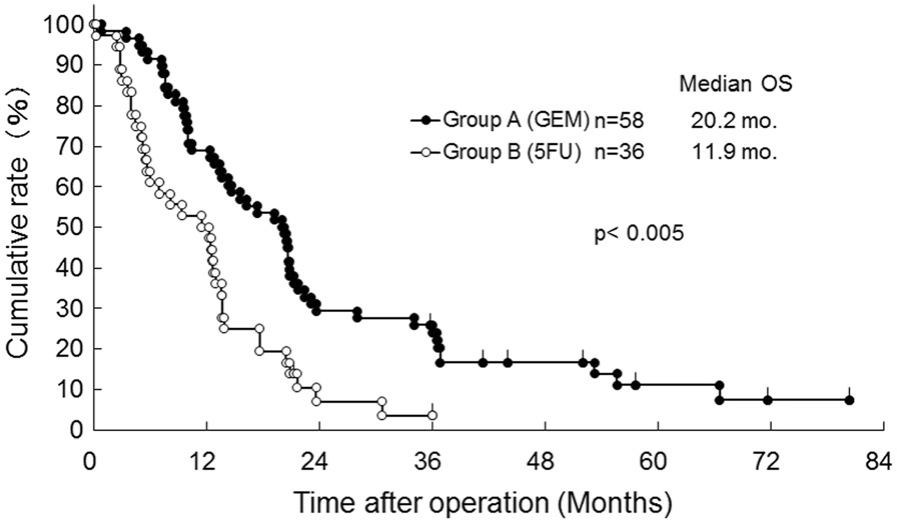
**Overall survival (OS) of resected pancreatic cancer patients who received adjuvant chemotherapy with bi-weekly gemcitabine (GEM: Group A) versus 5FU-based (5FU: Group B).** mo., months (Log-rank test *P*< 0.005).

**Figure 3:**
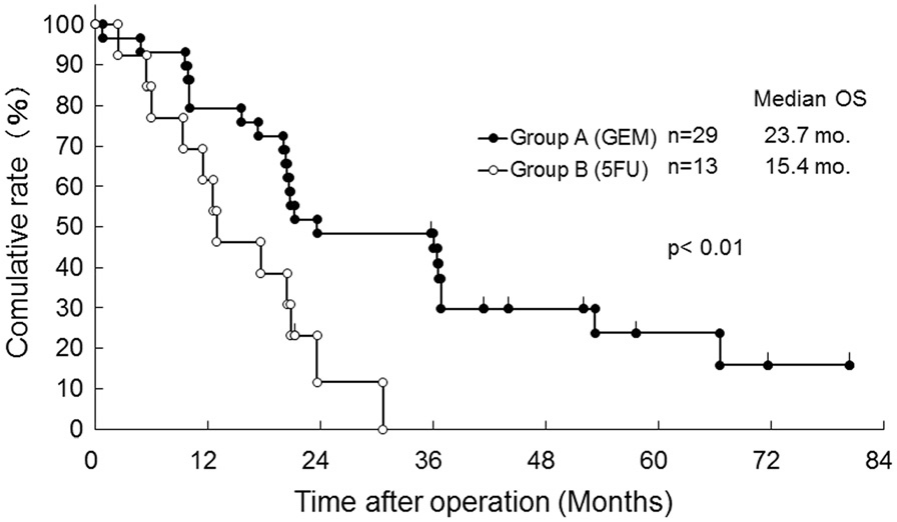
**Comparison of overall survival (OS) for resected pancreatic cancer patients between bi-weekly gemcitabine (GEM: Group A) and 5FU-based (5FU: Group B) in Stage 0–IIa.** mo., months (Log-rank test *P*< 0.01).

**Figure 4:**
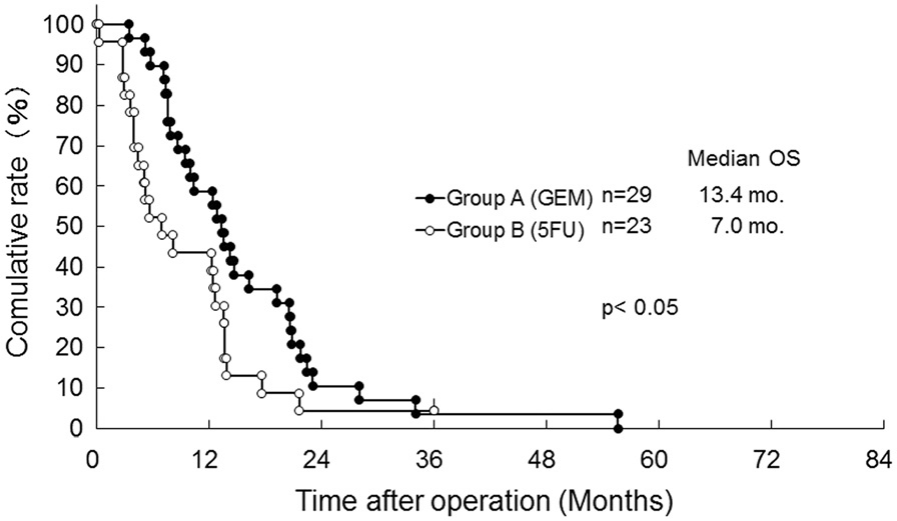
**Comparison of overall survival (OS) for resected pancreatic cancer patients between bi-weekly gemcitabine (GEM: Group A) and 5FU-based (5FU: Group B) in Stage IIb–IV.** mo., months (Log-rank test *P*< 0.05).

The median DFS of Group A was significantly improved in contrast with Group B (12.5 versus 6.6 months, *P* < 0.001, Figure 1). As shown in Figure 2, each median OS was 20.2 months in Group A versus 11.9 months in Group B, and each three-year OS rate was 24.0% in Group A versus 4.8% in Group B, suggesting that there was a significant difference between the two groups statistically (*P* < 0.005). When OS for patients at Stages 0–IIa and IIb–IV is compared between Groups A and B, a statistical difference was observed in each of the classified stages (Figures 3 and 4). The median survival of living patients was 52.1 months in Group A (*n* = 7) versus 28.7 months in Group B (*n* = 2). The longest survival time in Group A was over 6.7 years (80.4 months) while for Group B it was over 3.0 years (36.1 months). However, for the other patients, 51 in Group A and 34 in Group B died of recurrences of pancreatic cancer due to peritonitis carcinomatosa or multiple distant metastases in the liver, bone, and/or lung.

Univariate analysis for the subgroups in Table 3 showed that there were no statistical differences between the groups for 69-year-old or under females and T1–2 factors. As shown in Table 4, multivariate analysis for OS in both groups indicated three statistically important factors: bi-weekly adjuvant GEM chemotherapy, T2 or less, and R0.

**Table 3:** Overall survival in the total entire population and subgroups

			**Overall survival**			
	**Number**		**Median (months)**			
	**A**	**B**	**A**	**B**	***P*value**	
All patients	58	36	20.2	11.9	0.001	
Age ≦69	38	20	19.7	12.9	0.061	NS
>70	20	16	20.4	8.6	0.001	
Sex: male	36	20	20.4	6.3	0.001	
Sex: female	22	16	19.8	12.6	0.094	NS
T1–2	11	9	36.6	20.5	0.086	NS
T3–4	47	27	15.6	9.4	0.000	
N0	27	15	21.3	13.0	0.007	
N1	31	21	13.6	7.0	0.017	
R0	43	28	20.7	12.5	0.001	
R1	15	8	13.4	5.5	0.034	
Stage 0–IIA	29	13	23.7	15.4	0.008	
Stage IIB–IV	29	23	13.4	7.0	0.043	

*NS* no significant difference.

**Table 4:** Multivariate analysis for overall survival

	**HR (95% CI)**	***P *value**
Treatment with GEM	0.34 (0.20–0.57)	0.0001^a^
Age <70	1.09 (0.68–1.77)	0.7104
Male	0.89 (0.53–1.48)	0.6490
T factor ≦T2	0.49 (0.25–0.94)	0.0314^a^
N factor N0	0.75 (0.26–2.19)	0.6046
UICC stage ≦VIIA	1.23 (0.36–4.26)	0.7415
Resection status R0	0.38 (0.20–0.73)	0.0033^a^

^a^Statistically significant difference.

*CI* confidence interval; *GEM* gemcitabine; *HR* hazard ratio.

## Discussion

Several successful clinical trials of adjuvant chemotherapy based on 5FU and/or GEM for pancreatic cancer have been published [15-25]. Moreover, recent prospective, randomized studies of adjuvant GEM chemotherapy suggested an improvement in DFS and/or OS of pancreatic cancer patients [26-31], except for the ESPAC-3 trial [32]. Thus, adjuvant chemotherapy using GEM for pancreatic cancer has been recognized as an effective treatment. Although it was an adjuvant GEM trial, CONKO-001 indicated prolonged OS of pancreatic cancer patients and unsatisfied compliance (less than 62%) of GEM administration with the standard regimen was also reported [26]. In general, it has been thought that the main purpose of adjuvant chemotherapy is prolongation of DFS and/or OS by preventing or delaying disease relapse without impairing patients’ QOL. Previously, we had experienced that GEM administration with the standard regimen led to an unsatisfied compliance rate of 38% for pancreatic cancer patients after surgery. The patients suffered from severe side effects (over Grade 3) and had reduced QOL. Thus, the compliance rate generally seemed to be intimately correlated with patients’ QOL. Therefore, in terms of reducing toxicity, increasing compliance rate, and obtaining acceptable QOL, a phase I study was carried out in our department to determine an optimal dose-intensity of adjuvant GEM chemotherapy. After the phase I trial, in which the optimal dose-intensity was set at bi-weekly 1,000 mg/m^2^ of GEM administration, this trial studied whether the optimal, medium dose-intensity adjuvant bi-weekly GEM chemotherapy for pancreatic cancer patients would obtain both prolongation of OS and satisfactory compliance, that is, the patients’ QOL was studied to see if it was acceptable.

Results from this clinical study suggest that the adjuvant bi-weekly chemotherapy with 1,000 mg/m^2^ of GEM (Group A) statistically prolonged DFS and OS for pancreatic cancer patients when compared with the adjuvant 5FU-based chemotherapy (Group B) as a whole and in Stage 0–IIa and Stage IIb–IV subgroups. Thus, the adjuvant bi-weekly GEM chemotherapy was more effective than the 5FU-based treatment in resected pancreatic cancer patients even though most patients in Group B converted to the standard GEM chemotherapy due to the recurrence of pancreatic cancer. Previously, it had been reported in a Norwegian randomized study [2], ESPAC-1, and other trials [7-9,15,16] that adjuvant chemotherapies using 5FU for resectable pancreatic cancer led to significant survival benefit. On the other hand, results from a phase III trial conducted by the EORTC on the gastrointestinal tract cancer cooperative group and two Japanese adjuvant chemotherapy studies indicated that 5FU could not achieve such a survival advantage [5,33,34]. The RTOG 97–04 study indicated that GEM was superior to 5FU as an adjuvant chemoradiotherapy agent [27,28]. Results from this study also supported the potency of GEM compared to 5FU. The compliance rate of Group A in this study was higher than that of CONKO-001 study [26] in which GEM was given with the standard regimen (93% versus 62%). According to EORTC QLQ-C30 and QLQ-PAN26, the QOL of patients in Group A was much better than that of Group B, suggesting that our administration rate and dosage of GEM, that is, the medium dose-intensity, were tolerable and applicable even to patients with pancreatic cancer following major surgery. The results of the univariate analysis shown in Table 3 implied that the malignancy potential of the tumors was stronger in the younger generation, the effectiveness of gemcitabine was less in females than males, and for smaller tumors the surgical treatment had a greater affect than adjuvant chemotherapy. The multivariate analysis shown in Table 4 suggested that early detection of pancreatic cancer, good curability, and adjuvant chemotherapy with bi-weekly GEM were important to obtain a prolonged OS, such as that for the previous standard of GEM chemotherapy.

Currently, combination chemotherapy of GEM with new agents, such as capecitabine, erlotinib, or bevacizumab, has been used to try to improve the poor survival rate of patients with advanced pancreatic cancer [35-41]. More recently, combination therapies using peptide vaccines with GEM, which have achieved striking tumor reduction in advanced pancreatic cancer patients, have been reported [42-46]. Nowadays, ongoing adjuvant chemotherapy trials, such as JASPAC-01 (GEM versus S-1), ESPAC-4 (GEM versus GEM+capecitabine), CONKO-005 (GEM versus GEM+erlotinib), and CONKO-006 (GEM versus GEM+sorafenib) are under trial [47-54]. These new approaches may be able to obtain better prognosis in patients after curative resection of pancreatic cancer. However, it seems likely that good QOL is as important a factor for resected pancreatic cancer patients as prolongation of OS.

## Conclusions

In conclusion, the medium dose-intensity adjuvant chemotherapy with bi-weekly GEM led to improved OS with acceptable QOL in pancreatic cancer patients after surgery.

## Abbreviations

5FU: 5-fluorouracil; CI: Confidence interval; CONKO: Charité Onkologie; DFS: Disease-free survival; ECOG: Eastern Cooperative Oncology Group; EORTC: European Organisation for Research and Treatment of Cancer; ESPAC: European Study Group for Pancreatic Cancer; GEM: Gemcitabine; HR: Hazard ratio; IC: Informed consent; IRB: Institutional Review Board; mo.: Months; NS: No significant difference; OS: Overall survival; JASPAC: Japan Adjuvant Study Group of Pancreatic Cancer; POD: Postoperative days; QLQ-C30: Quality of life questionnaire – core 30; QLQ-PAN26: Quality of life questionnaire – pancreatic cancer module 26; QOL: Quality of life; RTOG: Radiation Therapy Oncology Group; SD: Standard deviation; UICC: Union Internationale Contre le Cancer.

## Competing interests

The authors declare that they have no competing interests.

## Authors’ contributions

YT designed and directed the study, and drafted the manuscript. YT, SY, RS, TU, and TN performed the majority of operations. HK, NO, RM, RI, and KS collected and analyzed the data. KY, who was involved in editing the manuscript and critically revising it, gave final approval of the version to be published. All authors read and approved the final manuscript.

## Authors’ information

The subspecialty of all authors is hepato-biliary-pancreatic surgery and surgical oncology. In particular, YT, SY, RS, and TU use adjuvant chemotherapy for patients with pancreatic cancer. YT and KY are highly skilled hepato-biliary-pancreatic surgeons, approved by the Japanese Society of Hepato-Biliary-Pancreatic Surgery. KY is Professor of Surgery and Chief of Gastrointestinal Surgery at the Jikei University School of Medicine, and President of the Japan Chapter of the American College of Surgeons.
